# Hepatocellular Carcinoma Complicated by Echinococcal Cyst: A Case Report

**DOI:** 10.3389/fsurg.2021.816501

**Published:** 2022-02-02

**Authors:** Jiwu Guo, Chenzhe Ma, Xuewen Song, Futian Tang, Lingyun Guo, Jie Mao, Yumin Li

**Affiliations:** ^1^Department of General Surgery, Lanzhou University Second Hospital, Lanzhou, China; ^2^Key Laboratory of Digestive System Tumors of Gansu, Lanzhou, China; ^3^The Second Clinical Medical College, Lanzhou University, Lanzhou, China; ^4^Department of Pathology, Lanzhou University Second Hospital, Lanzhou, China

**Keywords:** hepatocellular carcinoma, hepatic hydatid disease, hepatitis B virus, alpha-fetoprotein, case report

## Abstract

Hepatocellular carcinoma (HCC), combined with hepatic hydatid disease, is a rare clinical case, having certain specificity in clinical diagnosis and treatment. We report a case of HCC combined with hepatic hydatid disease treated in our clinic to arouse the attention of clinicians to the disease. A 54-year-old female patient was admitted to the clinic on October 31, 2016 because of “Intermittent upper abdominal pain and discomfort for 1 month.” Abdominal CT in the previous hospital showed liver space-occupying lesions, and hepatic hydatid disease should be considered. The patient had a history of hepatitis B virus (HBV) infection since childhood but has not received antiviral treatment. She did have a history of life in pastoral areas. Laboratory examination results were as follows: alpha-fetoprotein (AFP) 1,210 ng/ml, HBV DNA: 5.32E + 3 IU/ml. Casoni test was positive. Enhanced CT of abdomen suggestion was: malignant liver tumor, hepatic hydatid disease. Gastroscopy and colonoscopy showed no abnormalities. She underwent an operation on November 10, 2016. Segment 5, 8 of hepatic, echinococcus internal capsule, and cholecyst were all removed. She took albendazole (0.4 g/day) for 6 months and oral entecavir (0.5 mg/day) antiviral treatment for a long time after surgery. From May 2017 to October 2019, a total of 5 cycles of transarterial chemotherapy embolization (TACE) were performed. The patient underwent surgical treatment, followed by TACE, antiviral therapy, and sequential albendazole treatment. The AFP level increased significantly, but there was no obvious recurrence of HCC in imaging.

## Introduction

Hepatocellular carcinoma (HCC) is the most common liver malignant tumor. Hepatitis B virus (HBV) is the main cause of major hepatic disease, especially for HCC (1). Hepatic hydatid disease is a common zoonotic disease in pastoral areas (2). Humans are the intermediate host of hydatid disease, and the liver is the most common organ of infection (3). HCC, combined with hepatic hydatid disease, is a rare clinical case, having certain specificity in clinical diagnosis and treatment. Five-year follow-up data of a patient with HCC combined with hepatic hydatid disease treated in our group is summarized and reported to arouse the attention of clinicians to the disease and provide more information for the diagnosis, treatment, and research of this disease.

## Case Report

A 54-year-old female patient was admitted to Lanzhou University Second Hospital on October 31, 2016 because of “intermittent upper abdominal pain and discomfort for 1 month.” Symptoms became worse after eating greasy food. She did not receive any treatment before the admission. Because the above symptoms gradually worsened, the patient went to the local clinic. CT of the abdomen reveals space-occupying liver lesions, which was considered more as hepatic hydatid disease. For further diagnosis, the patient visited our hospital. The patient has been a carrier of HBV since childhood and has not been tested for HBV DNA or received antiviral treatment. At the same time, her mother and siblings are both HBV carriers, and she has a history of life in a pastoral area in her childhood. Appendectomy was performed because of acute appendicitis in 2009, and postoperative recovery was fine.

## Examination

**Figure 1:**
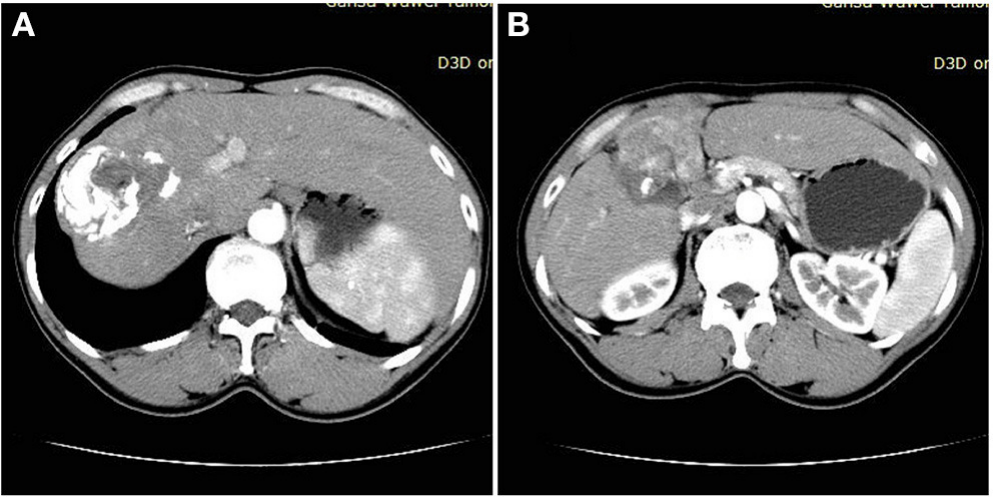
**(A)** Obvious calcification of liver space-occupying lesions. **(B)** Significant enhancement of liver space-occupying lesions.

Laboratory examination results were as follows: white blood cells (WBC) 3.7 × 10 ^9^ per L, red blood cells (RBC) 4.32 × 10^12^ per L, platelet (PLT) 119 × 10^9^ per L, T bilirubin (TBiL) 11.7 μmol/L (normal range: 3.8–25.8 μmol/L), direct bilirubin (DBiL) 5.4 μmol/L (0–6.8 μmol/L), alanine aminotransferase (ALT) 359 U/L (0–50 U/L), aspartate aminotransferase (AST) 200U/L (0–50 U/L). Alpha-fetoprotein (AFP) was 1,210 ng/ml (0–7 ng/ml). Test of HBsAg, HBeAg, Anti-HBe and Anti-HBc were positive, while test of Anti-HBs was negative. Level of HBV DNA was 5.32 × 10^3^ IU/ml (Normal <1.0 × 10^2^ IU/ml). Casoni test was positive. Enhanced CT of the abdomen (Figures 1A,B) suggested malignant liver tumor and hepatic hydatid disease, but no abnormalities were found in gastroscopy and colonoscopy.

The patient had a history of HBV infection and did not receive regular antiviral treatment, and AFP was significantly increased. Combined with imaging enhancement mode, she was diagnosed as having HCC and chronic HBV infection. In addition, considering the facts that the patient had a life history in a pastoral area, the Casoni test was positive, and the abdominal CT showed obvious calcification in the liver-occupying space, she was diagnosed as having hepatic hydatid disease.

**Figure 2:**
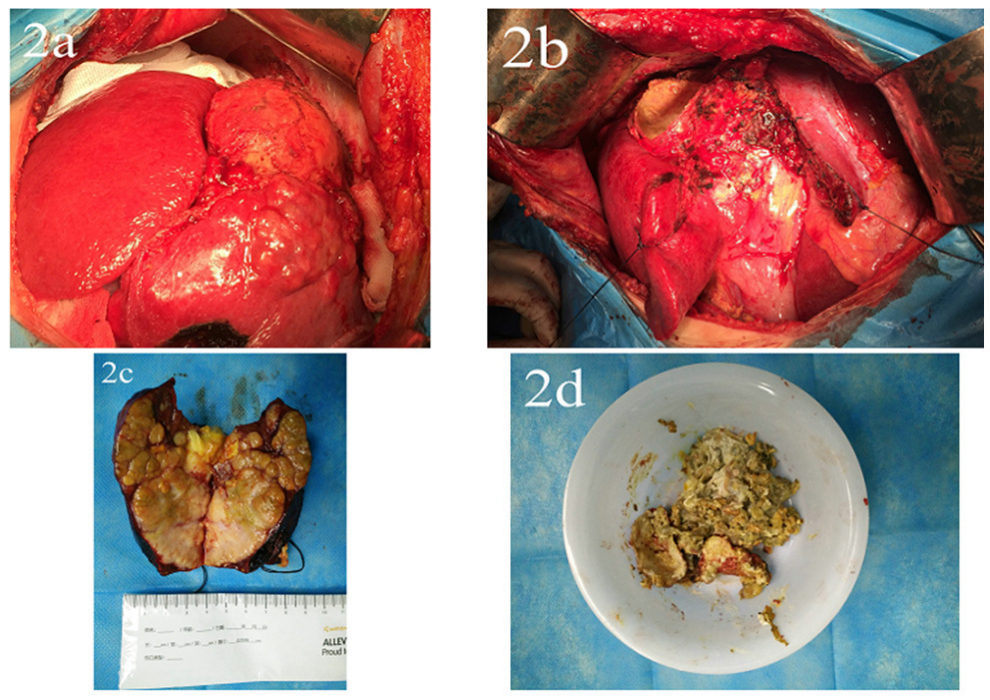
**(a)** Liver tumors and hydatid disease were seen in the right lobe of the liver during the operation. **(b)** Hepatic Segment 5, 8, hydatid internal capsule removal, and cholecystectomy were performed. **(c)** Liver tumor specimens. **(d)** Hepatic hydatid specimen.

**Figure 3:**
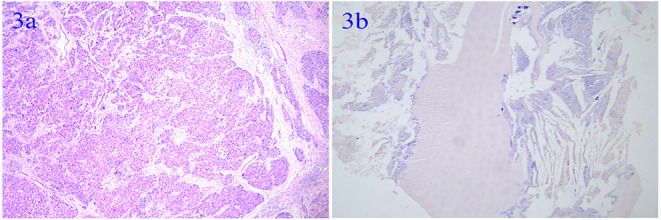
**(a)** Infiltrating moderately differentiated hepatocellular carcinoma of the liver. **(b)** Hydatid infection.

She underwent an operation on November 10, 2016. During the surgery, we found that the liver indicated signs of cirrhosis, and that the tumor and hydatid cysts were located in the right anterior lobe, adjacent to the right and middle hepatic veins. Therefore, hepatic Segment 5, 8 segment resection, hepatic hydatid internal capsule removal, and cholecystectomy (Figures 2a–d) were performed. She was discharged 8 days after the operation. Pathological results were infiltrating moderately differentiated hepatocellular carcinoma of the liver, with tumor thrombus in the vessel, accompanied by hydatid infection. Chronic cholecystitis was found without cancer invasion (Figures 3a,b). After being discharged from the hospital, she received long-term oral entecavir (0.5 mg/day) antiviral therapy and oral albendazole (0.4 g/day) for 6 months.

She underwent re-examination every 3 months after the surgery. In May 2017, the level of AFP increased to 895.5 ng/ml, and the first cycle of transcatheter arterial chemoembolization (TACE) treatment (oxaliplatin 150 mg + epirubicin 30 mg) was given. TACE was performed 3 times on June 27, August 7, and September 20, 2017, and AFP dropped to 3.73 ng/ml. In August 2019, AFP rose to 122.5 and 246.3 ng/ml in October 2019, but there was no evidence of recurrence of HCC in magnetic resonance imaging (MRI). The fifth TACE treatment was performed in October 2019, and there were no obvious signs of tumor staining during the TACE. AFP decreased to 182.7 ng/ml 1 month later. In March 2020, AFP elevated to 204.3 ng/ml, and the patient was advised to continue treatment, but she refused further treatment because of economic factors. In December 2020, AFP rose to 700.7 ng/ml. The recent follow-up was done in September 2021, and the patient was fine and received oral entecavir only.

## Discussion

Hepatocellular carcinoma (HCC) is a serious disease to human health and is widespread in East Asia; its occurrence is mainly related to HBV infection, drinking, obesity, and aflatoxin infection (4). Hydatid disease is a zoonotic disease and is more prevalent in developed agricultural and animal husbandry areas. The liver is the most common site of parasites and infections; besides, it was reported that the pancreas can also be involved (5). Because the hydatid disease could lead to a series of complications, it should be treated immediately (6, 7). Surgery and drug treatments are the main treatment methods (8–10).

After a comprehensive database search, we found that there are 25 patients with HCC combined with hepatic hydatid disease (11). This patient is the 26th case, and the follow-up time is the longest after the operation of all cases. As more and more cases were reported, the relationship between HCC and hydatid needs more attention. Romic conducted a systematic analysis of reported cases and classified these diseases into 5 types according to the location of tumors and hydatidosis (12), and this patient belongs to type I. More physicians believe that hydatid is a risk factor for HCC (13, 14). Bo et al. (15) performed a 1:5 case-control study and found that hydatid disease is a protective factor for the occurrence and development of HCC, but they did not analyze the synergistic effects of HBV infection. Therefore, the relationship and mechanism need to be further explored. During treatment of these diseases, misdiagnosis between hepatic hydatid disease and HCC occurs often, which needs more cautious when diagnosing (16). The patient survived for 5 years after surgery already, we believe that we can do more, but we failed for the patient's own factors.

## Conclusion

To sum up, HCC, combined with hepatic hydatid disease, is a rare clinical case. Therefore, such a case should be followed up and reported for more future research. During diagnosis and treatment of such patients, relevant examinations should be improved as much as possible, a decision should be made cautiously to reduce the occurrence of misdiagnosis, and a reasonable treatment plan should be formulated. Meanwhile, the relationship between echinococcosis and the occurrence, development, and prognosis of HCC needs further research.

## Data Availability Statement

The original contributions presented in the study are included in the article/supplementary material, further inquiries can be directed to the corresponding authors.

## Ethics Statement

Ethical review and approval was not required for the study on human participants in accordance with the local legislation and institutional requirements. The patients/participants provided their written informed consent to participate in this study. Written informed consent was obtained from the individual(s) for the publication of any potentially identifiable images or data included in this article.

## Author Contributions

JG, CM, and FT: article writing. XS: performed image acquisition. JG and LG: data collection. JG, JM, and YL: revised and improved the article. All authors contributed to the article and approved the submitted version.

## Funding

This work was supported by the National Natural Science Foundation of China (31770537); Gansu Administration of Traditional Chinese Medicine Project (GZK-2019-47); and Gansu Province College Innovation Fund Project (2021B-38).

## Conflict of Interest

The authors declare that the research was conducted in the absence of any commercial or financial relationships that could be construed as a potential conflict of interest.

## Publisher's Note

All claims expressed in this article are solely those of the authors and do not necessarily represent those of their affiliated organizations, or those of the publisher, the editors and the reviewers. Any product that may be evaluated in this article, or claim that may be made by its manufacturer, is not guaranteed or endorsed by the publisher.
